# The Influence of Wearables on Health Care Outcomes in Chronic Disease: Systematic Review

**DOI:** 10.2196/36690

**Published:** 2022-07-01

**Authors:** Graeme Mattison, Oliver Canfell, Doug Forrester, Chelsea Dobbins, Daniel Smith, Juha Töyräs, Clair Sullivan

**Affiliations:** 1 Queensland Digital Health Research Network Global Change Institute The University of Queensland Brisbane Australia; 2 Metro North Hospitals and Health Service Brisbane Australia; 3 Digital Health Cooperative Research Centre Australian Government Sydney Australia; 4 Centre for Health Services Research Faculty of Medicine The University of Queensland Brisbane Australia; 5 University of Queensland Business School Faculty of Business, Economics and Law The University of Queensland Brisbane Australia; 6 School of Information Technology and Electrical Engineering The University of Queensland Brisbane Australia; 7 Department of Applied Physics University of Eastern Finland Kuopio Finland; 8 Science Service Center Kuopio University Hospital Kuopio Finland

**Keywords:** wearable, chronic disease, health care outcome

## Abstract

**Background:**

Chronic diseases contribute to high rates of disability and mortality. Patient engagement in chronic disease self-management is an essential component of chronic disease models of health care. Wearables provide patient-centered health data in real time, which can help inform self-management decision-making. Despite the perceived benefits of wearables in improving chronic disease self-management, their influence on health care outcomes remains poorly understood.

**Objective:**

This review aimed to examine the influence of wearables on health care outcomes in individuals with chronic diseases through a systematic review of the literature.

**Methods:**

A narrative systematic review was conducted by searching 6 databases for randomized and observational studies published between January 1, 2016, and July 1, 2021, that included the use of a wearable intervention in a chronic disease group to assess its impact on a predefined outcome measure. These outcomes were defined as any influence on the patient or clinician experience, cost-effectiveness, or health care outcomes as a result of the wearable intervention. Data from the included studies were extracted based on 6 key themes, which formed the basis for a narrative qualitative synthesis. All outcomes were mapped against each component of the Quadruple Aim of health care. The guidelines of the PRISMA (Preferred Reporting Items for Systematic Reviews and Meta-Analyses) statement were followed in this study.

**Results:**

A total of 30 articles were included; studies reported 2446 participants (mean age: range 10.1-74.4 years), and the influence of 14 types of wearables on 18 chronic diseases was presented. The most studied chronic diseases were type 2 diabetes (4/30, 13%), Parkinson disease (3/30, 10%), and chronic lower back pain (3/30, 10%). The results were mixed when assessing the impact on a predefined primary outcome, with 50% (15/30) of studies finding a positive influence on the studied outcome and 50% (15/30) demonstrating a nil effect. There was a positive effect of 3D virtual reality systems on chronic pain in 7% (2/30) of studies that evaluated 2 distinct chronic pain syndromes. Mixed results were observed in influencing exercise capacity; weight; and biomarkers of disease, such as hemoglobin A_1c_, in diabetes. In total, 155 outcomes were studied. Most (139/155, 89.7%) addressed the *health care outcomes* component. This included pain (11/155, 7.5%), quality of life (7/155, 4.8%), and physical function (5/155, 3.4%). Approximately 7.7% (12/155) of outcome measures represented the *patient experience* component, with 1.3% (2/155) addressing the *clinician experience* and *cost*.

**Conclusions:**

Given their popularity and capability, wearables may play an integral role in chronic disease management. However, further research is required to generate a strong evidence base for safe and effective implementation.

**Trial Registration:**

PROSPERO International Prospective Register of Systematic Reviews CRD42021244562; https://www.crd.york.ac.uk/prospero/display_record.php?RecordID=244562

## Introduction

Chronic diseases account for 73% of deaths worldwide [1]. The World Health Organization categorizes chronic diseases into the following four main categories: cardiovascular diseases, cancers, chronic respiratory diseases, and diabetes [2]. Half of the people with chronic disease experience disability, which is defined as a limitation that restricts daily activities and lasts for at least 6 months. Disability results in increased dependence on social services [3] and reduced quality of life [4]. Chronic disease is responsible for a significant economic burden arising from direct health care costs and lost productivity because of illness and death. An estimated 36% of all allocated health care expenditure is directed at supporting individuals with chronic diseases [5].

Involving individuals with chronic diseases in active self-management programs is recognized as an essential component of chronic disease management [6-8]. Implementing self-management programs presents numerous challenges, including limited funding, awareness, and adherence to prescribed self-management strategies [9]. A key strategic priority area in the National Strategic Framework for Chronic Conditions of Australia [10] is active engagement; that is, providing a person-centered approach that puts people at the center of their own health care and empowers them to play an informed role according to their interests and abilities. The Quadruple Aim of health care [11] provides a structured model for an approach to health care that focuses on improving the individual experience of health care delivery by improving population health, minimizing health expenditure, and maintaining the well-being of health care providers. Implementing the principle of self-management into chronic disease programs to achieve the goals of the Quadruple Aim may optimize their intended outcomes.

Patient engagement is essential to satisfy the Quadruple Aim and promote self-management in chronic disease management. Part of this engagement process involves providing patients with autonomy over their own care, including active involvement in decision-making on treatment choices and healthy lifestyle changes. Commercially available technology, including wearable devices (*wearable*) and mobile apps, can provide users with feedback on their physiological parameters, which may give them more awareness of their condition [12]. Wearables are defined as sensory devices that can be attached to clothing or worn as an accessory, which allows the tracking of health information through a multitude of onboard sensors without hindrance [13]. Initially designed for the health and fitness industry to track wellness [14], most commercially available wearables can be used to monitor key health-related metrics, including heart rate, sleep quality, energy expenditure, and step count.

An evolving area of research is the integration of wearables into the support of individuals with chronic diseases by promoting self-management strategies [15,16]. Wearables unlock the capability to perform the continuous, real-time monitoring of health status [17]. This provides a comprehensive analysis of the individual’s overall health, which can be presented in a user-friendly format to patients and clinicians [18]. The ability to monitor health status remotely also strengthens integration into existing telehealth models of care, with the hypothetical capability of reducing in-person consultations between patients and clinicians [19].

Several validity studies have demonstrated early promise in the application of wearables for individuals with chronic diseases, including the prevention and treatment of cardiovascular disease [20], monitoring severity of Parkinson disease [21], and promoting adherence to exercise goals in diabetes mellitus and chronic obstructive pulmonary disease (COPD) [22]. However, implementing these devices into existing health care models will require strong empirical evidence supporting the efficacy of wearables on health care outcomes, clear implementation guidance, and research funding models [23]. Research on wearables in health care is currently in its infancy, with most studies adopting an observational research design, having small sample sizes, or focusing on healthy individuals [24]. There is minimal known, synthesized evidence for the influence of wearables on health care outcomes for individuals with chronic diseases.

To address this research gap, we conducted a systematic review addressing the question of the role of wearables in improving health care outcomes in chronic diseases. Our hypothesis is that a qualitative synthesis of the limited evidence to date may demonstrate early promise for wearables to positively influence health care outcomes, as defined by the Quadruple Aim. Our aim was to examine the influence of wearables on health care outcomes in patients with chronic diseases through a systematic review of the literature. The Quadruple Aim has been used to define health care outcomes as an internationally validated framework to guide approaches to improving health care service delivery. This research is relevant to health care researchers and clinicians exploring the capability of wearables in health care, as well as health system managers and the wearable technology industry.

## Methods

### Design

A systematic review design using qualitative methods was used. We adhered to the PRISMA (Preferred Reporting Items for Systematic Reviews and Meta-Analyses) statement [25]. Our PRISMA checklist is provided in Multimedia Appendix 1. This review was registered with the PROSPERO (International Prospective Register for Systematic Reviews) on April 22, 2021 (CRD42021244562).

### Search Strategy

A search of studies published between January 1, 2016, and July 1, 2021, was performed using PubMed, EMBASE, Web of Science, Scopus, CINAHL, and Cochrane Central Register of Controlled Trials. Studies published before 2016 were excluded to reflect the rate of technological advancement in the research and development of wearables [26]. Our strategy was developed in consultation with a medical research librarian. A combination of Medical Subject Headings (MeSH) terms and free text keyword terms was used, including *chronic disease* (MeSH), *wearable electronic devices* (MeSH), health care OR outcome*, and select chronic conditions such as *asthma*. Our full search strategy is available in Multimedia Appendix 2.

### Eligibility Criteria

Chronic disease is defined as any health condition lasting ≥3 months, which may lead to other health complications and may be associated with functional impairment or disability [27]. A health care outcome is defined as any parameter that demonstrates an effect on the patient experience, health care outcome (such as improvement in glycemic control in diabetes), clinician experience, or cost of health care delivery.

The inclusion criteria were (1) randomized controlled trials (RCTs) and observational studies, (2) feasibility studies observing the effect of wearables on a predefined health care outcome, and (3) studies published in peer-reviewed journals in English. Studies involving adults and children were included.

The exclusion criteria were (1) pregnant patient population, (2) studies demonstrating the validity or technological feasibility of wearables, (3) studies reporting the accuracy of wearables as their primary aim, (4) books or book chapters, (5) conference abstracts, and (6) systematic reviews.

### Screening

Screening of potentially eligible studies was performed in 3 steps: duplicate removal, title and abstract screening, and full-text screening. Duplicates were removed using EndNote (version 20; Clarivate) and Covidence [28]. Additional duplicates that were not removed during this process were removed manually. A total of 2 review authors (GM and DF) independently screened the titles and abstracts for inclusion using the predefined inclusion and exclusion criteria specified previously. All studies not discarded through this process were then screened via a full-text review process by 2 review authors (GM and OC), from which studies were identified for inclusion. Conflicts that could not be resolved between the 2 review authors were resolved by a third investigator (CS). Full data extraction, categorization, and labeling of papers were performed by 1 author (GM) and validated by a second author (OC).

### Risk of Bias Assessment

A risk of bias assessment was conducted for all RCTs by 1 author (GM). The Cochrane Collaboration’s tool for assessing the risk of bias [29] was used to assess each randomized study for bias from the randomization process, selection bias, bias because of missing outcome data, bias because of measurement of the outcome, and other biases otherwise not addressed. These are presented in Multimedia Appendix 3 [30-52]. For nonrandomized studies, the Risk of Bias in Nonrandomized Studies of Interventions tool [53] was used to assess bias because of confounding, selection bias, bias in classification of interventions, bias because of deviations from the intended interventions, bias because of missing data, bias in the measurement of outcomes, and selection of result bias. These are presented in Multimedia Appendix 4 [54-59].

### Data Extraction and Synthesis

All data were extracted from the identified studies under 6 key extraction themes that were most suited to address our original research question [60] (Textbox 1). A wide range of subheadings was selected, given the high variance in disease populations and outcome measures. A narrative qualitative synthesis of the included studies was conducted. The high heterogeneity of study designs, disease groups, patient populations, and outcome measures precluded the completion of the meta-analysis. Our results are based on the disease group, with relevant findings across all studies reported within the disease group in question. All outcomes were categorized as a component of patient experience, clinician experience, health care outcomes, or cost in alignment with the Quadruple Aim. A robust assessment of the synthesis is subsequently presented through critical reflection.

**Data extracted from included studies (adapted from Institute of Medicine standards [60]).**

**General information**

- Study ID, title, lead author contact details, citation, study funding sources, and country in which the study was conducted

**Characteristics**

- Aim, study design, start or end date, possible conflict of interest for authors, recruitment procedures used, population demographic, chronic disease studies, inclusion or exclusion criteria, and total number of participants

**Intervention and setting**

- Setting, intervention, cointervention (if any), control (if any), number of participants enrolled, number of participants in analysis, and number of withdrawals or exclusions lost to follow-up

**Outcome data**

- Unit of assessment, statistical analysis used, primary outcome (nature and measurement used), secondary outcomes (nature and measurements used), and length of follow-up

**Results of study analysis**

- Results with regards to primary, secondary, and exploratory outcome measures

**Additional information**

- Costs (if known), resources used (if known), and adverse events (if any)

## Results

### Classification of Wearables

The wearables used in these studies were designed to be worn either continuously or intermittently on the body. Their capabilities and commercial availabilities are summarized in Table 1. Of the 30 publications, 19 (63%) studied wearables placed on the waist (n=10, 53%) and wrist (n=9, 47%), with 3 (10%) publications studying wearables placed on multiple sites of the body. Multiple types of wearables were studied, including pedometers, smartbands, virtual and augmented reality (AR) systems, flash glucose monitoring systems, and intelligent shoe insoles.

**Table 1:** Wearable technology for the management of chronic diseases identified in studies within this review.

Site worn	Wearable technology	Chronic disease studied	Capability	Coexisting mobile app	Commercial availability
Arm	Inertial motion unit—shoulder and forearm placement [41]	Stroke	Movement feedback in upper limbs and estimation of angular displacement	No	No
Arm	SenseWear armband (model MF-SW) [55]	Progressive multiple sclerosis	Skin temperature measurement, limb motion detection, step count, and metabolic equivalents	No	No
Chest	Chest-worn wearable sensor [49]	Ischemic heart disease	Heart rate, respiratory rate, electrocardiogram measurement, and accelerometry	Yes	Yes
Foot	SurroSense Rx intelligent insole [44]	Diabetes mellitus with diabetic foot ulceration	Measurement of static plantar pressure	No	No
Hand	Kinesia—finger placement [50]	Parkinson disease	Feedback on finger movement	No	No
Head	EaseVRx HMD^a^—3D applied VR^b^ [35]	Chronic lower back pain	3D VR delivery	No	No
Head	Oculus Rift HMD [37]	Chronic neuropathic pain after spinal cord injury	3D VR delivery	No	Yes
Head	Google Glass [57]	Parkinson disease	Augmented reality	No	Yes
Multiple	Oculus Rift HMD and OptiTrack V120—motion-tracking devices [60]	Cerebral palsy and developmental dyspraxia	VR delivery and linear path tracking	No	No
Multiple	Gamepad (wearable 6-inertial sensor system) [52]	Parkinson disease	Visual feedback on movement	No	No
Multiple	Digital medicine offering—ingestible sensor and smart patch [43]	Hypertension and diabetes mellitus	Medication ingestion adherence	Yes	No
Waist	Pedometer (unspecified) [34]	Multiple chronic diseases	Step count	No	Yes
Waist	Yamax DigiWalker CW-701 pedometer [33]	Chronic lower back pain	Step count	No	Yes
Waist	Fitbit Zip [38,40,56]	COPD^c^, rheumatoid arthritis, and type 2 diabetes	Step count and calories	No	Yes
Waist	Omron HJ-321 pedometer [48]	COPD	Step count	No	Yes
Waist	Omron HJ-720ITC pedometer [31]	Juvenile idiopathic arthritis	Step count	No	Yes
Waist	Coffee WALKIE+Dv3 pedometer [54]	Metabolic syndrome	Step count	No	Yes
Waist	Omron HJ-112 [45,59]	Obesity with multimorbidity	Step count	No	Yes
Wrist	Fitbit Charge [46]	Type 2 diabetes mellitus	Step count and calories	Yes	Yes
Wrist	Fitbit Charge HR [39]	Advanced liver disease	Step count, heart rate, and calories	Yes	Yes
Wrist	Fitbit Flex [48]	Osteoarthritis (knee)	Step count, distance, calories, and sleep stage estimation	Yes	Yes
Wrist	Heart Rate Smart Wristband GSH405-B6 [61]	Chronic kidney disease	Step count, calories, and sleep stage estimation	Yes	Yes
Wrist	Fitbit Charge 2 [58]	Osteoarthritis	Step count, calories, heart rate, floors climbed	Yes	Yes
Wrist	Fitbit Flex 2 [36]	Rheumatoid arthritis and systemic lupus erythematosus	Step count, calories, and sleep stage estimation	Yes	Yes
Wrist	Nike+ FuelBand [51]	Peripheral vascular disease	Step count and calories	Yes	Yes
Wrist	Samsung Charm [32]	Obstructive sleep apnea	Step count, calories, and distance	Yes	Yes

^a^HMD: head-mounted display.

^b^VR: virtual reality.

^c^COPD: chronic obstructive pulmonary disease.

### Study Selection

**Figure 1:**
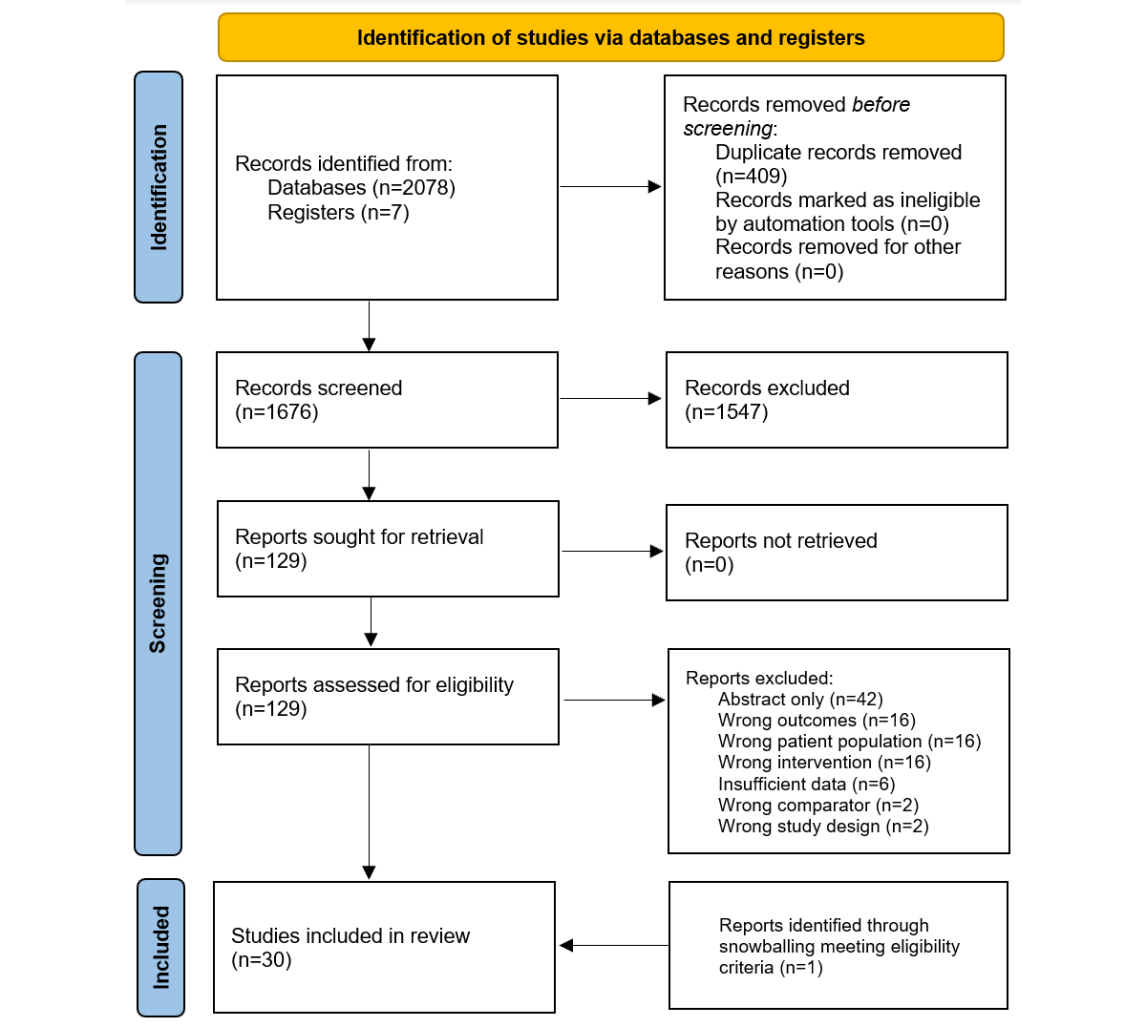
PRISMA (Preferred Reporting Items for Systematic Reviews and Meta-Analyses) flowchart outlining the study selection sequence.

The study selection sequence is outlined in a PRISMA flowchart, which is presented in Figure 1. Our search yielded 2078 articles, with a further 7 articles identified through snowballing. From a total of 2085 articles, 409 (19.62%) were identified as duplicates and were thus removed, and a further 1576 (75.59%) studies were excluded after screening abstracts between July and August 2021, leaving 129 (6.19%) studies assessed for eligibility via full-text review, of which 99 (4.75%) were excluded. One study was identified through snowballing. A total of 31 studies met the inclusion criteria. Following peer review, 1 study was removed, leaving 30 studies included in our qualitative synthesis.

### Study Characteristics

Multimedia Appendix 5 [30-52,54-59] presents the characteristics of all included studies. A total of 2446 participants across 9 countries were included. These participants had a mean age ranging from 10.1 to 74.4 years and 56.42% (1380/2446) were female. Of the 30 studies, 2 (7%) targeted a pediatric population (aged<18 years [30,31], 21 (70%) studied adults with a mean age between 40 and 65 years [32-49,54,55,61], and 6 (20%) evaluated individuals aged >65 years [50-52,56-58]. Approximately 50% (15/30) of studies recruited participants from specialist tertiary clinics, with the remaining 50% (15/30) recruiting participants in the community (eg, primary care settings and rehabilitation centers). Of the 30 studies, 24 (80%) were randomized, with the remaining 6 (20%) using a nonrandomized methodology. All randomized studies were subject to a risk of bias in the blinding of participants because of the nature of the wearable intervention being present or absent, with a notable exception being the use of sham virtual reality (VR) headsets in a study, which used identical hardware with different software with which the user interacted [35].

### Chronic Disease Management

Within the literature synthesized, wearables and their influence on health care outcomes in 8 disease systems were studied across 18 chronic diseases. There were predominantly mixed findings within the studies included in this review, which are summarized in the following sections.

#### Stroke and Neurological Disease

##### Stroke

Lin et al [41] explored the capability of inertial measurement units (IMUs) in detecting full-body human motion in 20 participants recovering from stroke and assessed the effect of physical activity sessions 3 times per week on upper limb neurological recovery guided by IMUs compared with conventional rehabilitation across 6 weeks. In this study, all 5 Fugl-Meyer assessment (an outcome measure for sensorimotor stroke recovery [62]) subscores improved in both arms, with the deviation angle of shoulder extension rotation during shoulder abduction substantially improving in the IMU group (*P*=.02) but not in the control group.

##### Neurological Disease

Approximately 10% (3/30) of studies [50,52,57] explored the effect of a range of wearable systems on motor assessment scoring, balance, self-selected gait speed, and symptom severity scoring in Parkinson disease. These were all perceived as acceptable and enjoyable to use. However, the only positive outcome was the influence of a wearable 6-inertial system on balance and self-selected gait speed when compared with conventional physiotherapy [52]. VR [30,37] and AR [57] systems had mixed results on their desired primary outcomes, although a positive effect was observed in 3D VR interfaces on chronic neuropathic pain after spinal cord injury when compared with sham VR. The reviewed study on the impact of VR on neuropathic pain was limited in size, with only 17 participants being recruited. However, a similar and larger study that incorporated 188 participants demonstrated a positive impact on symptoms in people with chronic lower back pain [35].

#### Rheumatological and Musculoskeletal Disease

##### Chronic Lower Back Pain

Chronic lower back pain was studied in 10% (3/30) of publications, with a total of 430 adult participants. Both Amorim et al [42] and Lang et al [33] used commercially available wearables to observe their effects on care-seeking episodes [42] and perceived disability [33], respectively, when compared with usual care. They found no influence on the studied primary outcome. Amorim et al [42] also observed a statistically significant increase of 183.1 min/week in walking time using a Fitbit wearable. However, this did not affect the number of care-seeking episodes in this group. In contrast, Garcia et al [35] studied subjective pain scoring following the implementation of a 3D VR headset (incorporating cognitive behavioral therapy and mindfulness practices) compared with the use of a 2D sham VR system. They found a positive influence of the 3D VR system on subjective pain scoring in addition to secondary outcome measures of pain inference on perceived physical activity, mood, sleep, and stress levels.

##### Inflammatory Arthritis

Measurement of physical activity was observed in 7% (2/30) of studies that involved people with chronic inflammatory arthritis [31,36], with contrasting results. Blitz et al [31] found a positive influence on the 6-minute walk test (6MWT) in adolescents with juvenile idiopathic arthritis with lower extremity involvement through the use of pedometers and guided education when compared with a pedometer group without education. Li et al [36] found no effect on the moderate to vigorous activity time in people with either rheumatoid arthritis or systemic lupus erythematosus when using a Fitbit Flex 2 compared with people who received usual care. The use of a pedometer coupled with guided education was demonstrated to positively affect subjective fatigue in rheumatoid arthritis in a study by Katz et al [38], who also noted a statistically significant increase in step count in the pedometer group.

##### Osteoarthritis

Both Zaslavsky et al [58] and Smith et al [47] studied the effect of Fitbit wearables on both sleep quality [58] and exercise capacity (6MWT) [63] in osteoarthritis. Both interventions involved the use of a Fitbit device combined with motivational outputs based on the data provided by the wearable. The results were mixed; Zaslavsky et al [58] found a positive effect of Fitbit-guided exercise on subjective sleep quality, whereas Smith et al [47] did not demonstrate a difference in the 6MWT between the Fitbit and non-Fitbit groups. Sleep quality outcomes were similarly mixed, with improved subjective scoring of sleep quality. However, the objective sleep quality assessed using wrist actigraphy was not affected.

#### Respiratory Disease

##### COPD Management

Pulmonary rehabilitation (PR) is a critical component of the nonpharmacological management of COPD [63]. Approximately 7% (2/30) of publications studied the effects of pedometer-guided exercise on adherence to exercise targets [56] and the 6MWT [48] in 70 participants with stable COPD. Ward et al [56] demonstrated 53% adherence to prescribed step count targets with a 20% increase in total step count from week 1 to week 6 of exercise and observed statistically significant improvements in subjective dyspnea, emotional functioning, and disease mastery. Widyastuti et al [48] noted an improvement in the 6MWT using a pedometer from the start of PR to completion; however, this was not greater than the improvements noted in the control group.

##### Obstructive Sleep Apnea

A randomized, 3-armed study performed by Kim et al [32] recruited 60 individuals with clinician-diagnosed obstructive sleep apnea to assess the effects of a mobile app (MyHealthKeeper) and wearable (Samsung Charm) on weight reduction across 4 weeks compared with an app-only group and an education-only group. They observed significant weight loss in both the app and wearable group (mean weight loss 1.4 kg; *P*=.02) and app-only group (mean weight loss 2.0 kg; *P*=.02), which did not translate into secondary outcome measures addressing symptom scoring (snoring frequency, daytime sleepiness, and witnessed apnea).

#### Cardiovascular Disease: Ischemic Heart Disease

Maddison et al [49] performed a randomized pilot study in 2019 comparing cardiac telerehabilitation with center-based programs for adults with coronary heart disease. Participants were randomized to either a telerehabilitation group—comprising exercise coaching and monitoring using a chest-worn sensor—or a conventional cardiac rehabilitation group across 12 weeks. The chest-worn sensor allowed real-time monitoring of heart rate, respiratory rate, electrocardiogram, and accelerometry during rehabilitation. The primary outcome measure studied was maximal oxygen consumption (VO_2_ max), which refers to the maximum amount of oxygen that an individual can use during maximal exercise and is used as a marker of cardiovascular fitness. VO_2_ max was comparable in both groups at 12 weeks, demonstrating noninferiority of telerehabilitation compared with center-based rehabilitation (adjusted mean difference VO_2_ max 0.51, 95% CI –0.97 to –1.98 ml/kg/min; *P*=.48).

#### Endocrine Disease: Type 2 Diabetes Mellitus

Abbott et al [44] studied an intelligent wearable insole (SurroSense Dx) and its ability to prevent diabetic foot ulceration in 58 people with diabetes who recovered from prior foot ulceration. The insole detects high-pressure areas and, in the intervention group, feeds this information back via an integrated app to offload pressure on the affected area. The study did not demonstrate a reduction in the number of diabetic foot ulcer episodes between the groups, despite good adherence to the technology use.

The influence of step count and goal setting on hemoglobin A_1c_ (HbA_1c_) levels in type 2 diabetes mellitus was studied by both Kooiman et al [40] (using a Fitbit Zip pedometer and web-based self-tracking program) and Lystrup et al [46] (using a Fitbit Charge smartwatch and web-based leaderboards). Both studies reported no significant differences in HbA_1c_ levels after these interventions.

A multifaceted wearable system was studied by Frias et al [43] for people with both diabetes and uncontrolled hypertension comprising an ingestible sensor and an accompanying patch used to detect adherence to prescribed medication in a digital medicine offering. They observed a significant reduction in systolic blood pressure at 4 weeks as the primary outcome of the study, as well as a reduction in systolic blood pressure at 12 weeks, HbA_1c_, fasting blood glucose, and serum low-density lipoprotein as secondary outcome measures.

#### Obesity and Metabolic Syndrome

Takahashi et al [45] performed an RCT to determine the effect of pedometer use and behavioral goal setting compared with counseling on exercise and nutrition, step count, gait speed, and grip strength in adults who were overweight and obese with multimorbidities (defined as having>6 comorbid medical conditions). There was no significant improvement in step count between the groups, and although the pedometer and goal-setting groups demonstrated statistically higher grip strength (1 kg; *P*=.01) at 4 months, the clinical importance of this improvement in strength is uncertain. A secondary analysis of this study [59] found no significant differences in within-group weight loss.

Huh et al [54] explored the potential of applying a Coffee WALKIE pedometer with an accompanying mobile app for metabolic syndrome management. They recruited 53 participants with metabolic syndrome and observed the daily step count, calorie expenditure, and proportion of resolved cases of metabolic syndrome following a 12-week study period. Only 20 participants completed the study, and 32 reported communication issues between the wearable and mobile apps, thus leading to withdrawal; however, they observed a mean reduction in systolic blood pressure (mean percentage reduction of 6.71%) and diastolic blood pressure (mean percentage reduction of 7.98%) leading to resolution of metabolic syndrome in 9 participants (*P=.*02).

#### Chronic Kidney Disease

Li et al [61] studied the ability of a health management platform, incorporating a smart wristband (Heart Rate Smart Wristband GSH405-B6) and accompanying app (WowGoHealth app) to improve participants’ self-management abilities and delay the progression of renal decline in chronic kidney disease. All 60 participants were adults, had clinician-diagnosed chronic kidney disease (stages 1 to 4), and were randomized into either the health management platform group or usual care group for a period of 90 days. The intervention group had significantly higher self-efficacy and self-management scores at the end of the study period. The mean kidney disease–related quality of life scores were also significantly higher in the intervention group than in the control group. This translated into reduced rates of renal decline, with a significantly slower decline in estimated glomerular filtration rate observed in the intervention group (–0.56 mL/min/1.73 m^2^) than control (–4.58 mL/min/1.73 m^2^).

#### Liver Disease

Chen et al [39] evaluated the impact of a home-based physical activity program on physical fitness using personal activity trackers (Fitbit Charge HR) to remotely monitor and guide exercise efforts in people with advanced liver disease. All 20 participants were provided with a high protein and amino acid diet as a separate exploratory outcome of the study. Physical fitness was assessed using the 6MWT, and computed tomography–based anthropometry was used to assess skeletal muscle volume. The study found a statistically significant improvement in the 6MWT in the home-based physical activity program group versus the control group, with a between-group walking distance difference of 151 m. This did not translate into differences in the computed tomography–based anthropometrics or quality of life outcome measures.

#### Peripheral Vascular Disease

Normahani et al [51] set out to determine whether the use of a feedback-enabled wearable could improve walking distance and quality of life in people with peripheral vascular disease and intermittent claudication. A total of 37 participants were randomized into an intervention group to use a Nike+ FuelBand with access to data via mobile device pairing and a computer or a control group who received usual care. After 6 months, participants in the Nike+ FuelBand group almost doubled their median maximal walking distance (MWD) from baseline (178 m vs 80 m), and this was sustained at 12 months. Statistically significant improvements were also observed in the distance to the onset of claudication, with this distance improving by 75 m from the baseline at 6 months (112 m vs 40 m). Participants in the control group did not display improvements in MWD or distance to claudication onset. An improvement in Vascular Quality of Life Questionnaire scores at 12 months was also observed in the Nike+ FuelBand group, which correlated with improvements in the MWD and claudication distance.

### Theoretical Aspects of Wearables in Improving Health Care Outcomes

Of the 30 studies, 15 (50%) found a positive impact of wearables on the primary outcome studied. These findings were observed in studies involving multiple chronic disease systems and using multiple wearables. Wearables can be used to deliver nonpharmacological therapy to improve subjective pain scoring, as demonstrated by both Austin et al [37] and Garcia et al [35] in using 3D VR systems to deliver cognitive behavioral therapy and mindfulness practices to users with chronic pain. Tunur et al [59] found a lack of effect in delivering therapy through AR-based dance interventions in Parkinson disease, although the outcome studied was motor assessment, and only 7 participants were enrolled in the study.

Approximately 37% (11/30) of studies observed the capability of wearables to encourage and improve physical activity. There was no uniform measurement system to quantify physical activity across the included studies, with the 6MWT [31,39,47,48], 10-minute walk test [52], minutes of moderate to vigorous physical activity time [34,36], step count [45], MWD [51], VO_2_ max [49], and adherence to prescribed walking therapy [56] used to measure exercise capacity. A positive impact was observed in 17% (5/30) of studies assessing the influence of wearables on physical activity. There did not appear to be a strong relationship between wearables and improvement in physical activity in these studies.

Approximately 20% (6/30) of studies [33,35,37,38,58,61] used subjective scoring systems to assess the influence of the studied wearables on primary outcome measures. Of these 6 studies, 5 (83%) [35,37,38,58,61] found a positive effect on the primary outcome across multiple chronic diseases. Given the potential for wearables to empower users with additional health-related data, subjective scoring systems may improve significantly using wearables, which may be reflected in health-related quality of life assessments. However, further research is required in this area.

### Associations Between Wearables and Outcome Measures

Several associations were noted between the types of wearables used and the studied outcomes. One such observation was the positive effect of 3D VR systems on pain scoring in 2 distinct chronic pain syndromes [35,37], in which both studies used sham VR as the comparator. Both publications reported minimal side effects of using these immersive systems, with specialized programs used to counter the possible effects of cybersickness (motion sickness specific to the use of VR headsets). The use of either pedometers or smartbands (eg, Fitbit devices) had mixed effects on exercise capacity, with 10% (3/30) of studies [31,39,51] reporting improvements in walking distance using these devices and 7% (2/30) of studies [48,63] finding no influence on this outcome. Mixed results were also observed when studying the influence of either pedometers or smartbands on weight. Kim et al [32] found a significant reduction in BMI through the use of a Samsung Charm fitness tracker in people with obstructive sleep apnea. This is in contrast with the findings of both Lystrup et al [46] (using a Fitbit Charge) and Takahashi et al [63] (using a pedometer).

### Mapping Outcomes to the Quadruple Aim of Health Care

A total of 155 outcome measures were studied within the included studies. Of these 155 outcomes, 139 (89.7%) addressed the *health care outcomes* component of the Quadruple Aim, with 12 (7.7%) representing the *patient experience* of using wearables. Approximately 1.3% (2/155) of outcome measures evaluated the *clinician experiences* of wearables, with the remaining 1.3% (2/155) addressing *cost*. Within the health care outcomes, the most frequently studied included pain (11/155, 7.1%), quality of life (7/155, 4.5%), step count (7/155, 4.5%), and physical function (5/155, 3.2%). Approximately 7% (2/30) of studies explored the impact of wearables on patient experience as their primary outcome [51,57]. All outcome measures mapped to the patient experience included acceptance of the technology [57], adherence to wearing the device [36,44], compliance [50], engagement [35], presence (using VR) [37], satisfaction [31,35] and usability [50]. A summary of all outcomes in the included studies is presented in Multimedia Appendix 6 [30-52,54-59].

There were no studies in which the primary outcome could be classified as addressing either the clinician experience or cost of health care provision, with only 4 secondary or exploratory outcomes representing these facets of the Quadruple Aim. In the management of Parkinson disease, it was noted that when using data generated by a finger-worn wearable, the consultation time spent with a neurologist was significantly reduced when compared with an in-person clinical assessment, although this was reported with a statistical statement of inequality (range 29-45 minutes vs 45-60 minutes; *P*<.05) [50]. Widyastuti et al [48] performed a cost-effectiveness analysis of pedometer-guided PR in COPD compared with conventional center-based PR and found that the cost of establishing pedometer-guided care was significantly cheaper than that of conventional PR, with a mean cost-saving of €76.3 (US $80.30) per patient. Maddison et al [49] also performed a cost-effectiveness analysis of telerehabilitation versus center-based rehabilitation, reporting a per capita program delivery difference of £1185 (US $1247.10; *P=*.02); however, hospital service use costs were not significantly different (*P*=.20).

### Robust Assessment of the Narrative Qualitative Synthesis

A critical reflection of the synthesis process was performed [64]. The methodology used for this synthesis was aligned with the PRISMA guidelines and registered with PROSPERO, and data extraction was performed based on 6 main themes. It was believed that this enabled researchers to answer the predefined research question. Although a wide range of chronic diseases and wearables were studied and included in our synthesis, certain patient populations were not represented, including those with underlying mental health conditions, chronic skin conditions, or malignancy. This may reflect a limitation of our search strategy or may equally reflect a lack of understanding of the use of wearables for certain disease groups.

The evidence used was not subject to major bias. Most publications involved a relatively small number of participants, and only 80% (24/30) of the studies were randomized. No assumptions were made regarding adherence, acceptability, intended use, or anticipated outcomes in the included studies. No discrepancies were identified within the included studies, and focus areas of future research are highlighted in the *Discussion* section. The strict search criteria precluded the identification of ongoing studies. However, by reviewing the many trial protocols in our abstract screening and review of the gray literature, it is anticipated that the rate of technological advancement and interest in the field will strengthen the existing evidence base in wearables and health care outcomes in chronic disease management. The aspects that may influence the implementation of wearables in existing health care platforms are highlighted in the *Discussion* section.

## Discussion

### Principal Findings

A total of 30 studies published between 2016 and 2021 investigated the effectiveness of wearables in improving health care outcomes in individuals with chronic diseases. Our systematic review found both positive and neutral results when studying the influence of wearables on health care outcomes in chronic disease. Encouragingly, none of the identified studies demonstrated a negative impact of wearables on outcomes, such as adverse effects, treatment burden, or the inability of study participants to effectively use the prescribed technology because of poor inadequate digital literacy.

We identified several studies demonstrating the positive influence of the studied wearables on primary health care outcomes. One such example is the positive influence of guided exercise prescription using the Nike+ FuelBand on peripheral vascular disease with intermittent claudication [51]. Approximately 7% (2/30) of RCTs found a significant improvement in quality of life indices [51,61], which correlated with either greater self-efficacy in chronic disease management [61] or functional capacity [51].

A key finding was the lack of a clear association between the use of a particular wearable and its acceptability within a chronic disease population. Most health apps synchronizing with wearables, including those studied in this review, are disease focused and provide information on the studied conditions, such as pressure points in diabetic foot ulceration. Although this does provide clinically useful information, it is unclear whether patients use this information effectively to guide their self-management or even find it useful. We also have limited knowledge of the acceptability of wearables to patients with chronic disease or whether there are any barriers to implementation within specific chronic disease groups. Supporting mobile apps should be designed using a patient-centered approach, incorporating personalized advice and recommendations from the health data provided by the accompanying wearable. This may unlock the potential of wearables in chronic diseases.

To rationalize the incorporation of wearables into existing health care models, it is important to demonstrate cost-effectiveness; however, economic outcomes were not incorporated into the publications identified in this review. Wearables enable remote monitoring, which has its own economic advantages. One of the studies involving patients with implantable cardiac defibrillators found that remote monitoring reduced health care costs by 25% [65]. Transferring even a small proportion of *center-based* monitoring services established for people with chronic disease remotely through the use of wearables has the potential to substantially reduce the cost of care delivery in many chronic disease settings. Robust economic analyses incorporating the number needed to treat analyses should be inherent to future studies on wearable technologies.

### Implications for Clinical Practice

There are several challenges in the implementation of wearables in health care. Wearables were initially designed for the health and fitness industry and are not subject to the regulatory standards required for medical equipment. Only a handful of wearable technologies have been approved by the US Food and Drug Administration, such as the Apple Watch Series 6 electrocardiogram app for detecting atrial fibrillation [66]. Most commercially available wearables are classified as nonmedical devices, and although some software within these are accurate, they cannot support clinical decision-making without legal regulation [67]. We noted that 73% (22/30) of studies included in this systematic review used commercially available wearable technology that is not currently legislated under either the US Food and Drug Administration or the Therapeutic Goods Administration (Australia); therefore, this may represent a limitation in applicability to health care settings. Wearables can also collect many different aspects of user information using sensor technology, which requires stringent data security and privacy processes. The accuracy of recently implemented software in wearables is variable and must be recognized to prevent avoidable misdiagnosis and unnecessary patient-clinician interfaces and minimize patient anxiety [68]. Until these challenges are clearly addressed, it is difficult to envision the safe implementation of wearables in existing health care models.

There is a high degree of variance in the chronic diseases studied and in the wearables used, which limits the ability to provide a strong evidence base to support the use of a specific wearable to treat a specific cause. Further research needs to focus on the impact of a specific wearable for a specific chronic disease to generate evidence for its use, especially given the multiple capabilities of most wearables. In addition, there may be several applications within the studied wearables for which only one wearable is particularly effective for a chronic disease group. Understanding these capabilities through targeted research is essential for the implementation of wearables in chronic disease management.

A finding from this systematic review was the lack of studies focusing on the clinician experience of wearables in health care. Without a deep understanding of the perceived benefits and risks of wearables in chronic disease management from the clinician’s perspective, it is difficult to envision their integration into clinical practice. Challenges to more established digital health transformative initiatives, such as introducing electronic health records, likely exist for the use of wearables in health care. These include changes to clinical workflows, patient safety concerns, learning curves of practitioners in understanding new technology, and challenges in integrating systems [69]. Interdisciplinary teams, including clinicians, data scientists, and experts in artificial intelligence, will be required to shape the future of wearables in health care because of the limited familiarity of clinicians with big data and artificial intelligence [70]. Alongside observational and randomized controlled quantitative analyses, qualitative research into the clinician’s perspective will prove invaluable in optimizing the use of wearables in health care.

### Strengths and Limitations of This Review

There were several strengths of our review. Given the rapid shift to care decentralization amidst a global pandemic [71], this review provides a foundational evidence base for the effect of wearables in improving health care outcomes for individuals with chronic diseases. A large number of participants was obtained, encompassing a range of chronic diseases, with a wide mean age range and reasonably equal gender distribution. Approximately 80% (24/30) of studies implemented a randomized controlled design, generating a comparator to assess the effect of the wearable intervention studied, which was predominantly the gold standard of care for the studied chronic disease. Although pedometers have been outdated commercially by newer technology, their inclusion as wearables in this study remains a strength because of the long-term evidence of their ability to encourage physical activity [72].

Our review has several limitations, such as the use of now outdated wearables. All smartwatch brands used in these studies have since been superseded by more advanced devices with greater technical capabilities. Although this may simply reflect the rate of technological advancement, it may also indicate that the studies published in this review may not reflect the capabilities of wearable technology from 2021 onward. It is unknown whether advanced information, including heart rate variability, Firstbeat algorithms such as stress levels and Garmin’s Body Battery, and advanced sleep stage estimation may infer real-time information on disease status. The rate of increasing technical capability of wearables justifies the regular systematic review of the literature, given the increasing publication outputs and commercial availability of more advanced wearables.

The interventions used as comparators differed greatly between studies, which made the comparison of the results challenging. The heterogeneity of the participants in each study precluded the ability to perform a meta-analysis, leading to an inability to assess the strength of evidence for a particular wearable on a predefined health care outcome. Although there were no apparent major risks of bias in our risk of bias assessments, this process was conducted by only 1 researcher. In addition, within the included studies, there was a lack of multicenter trials, which we propose should be conducted to increase the statistical robustness. All outcome measures reported were quantitative in nature, which represents a limitation, as qualitative research strengthens the existing evidence base. Given the increasing number of publications in this field, it is highly likely that a meta-analysis will be feasible for future systematic reviews of this nature. In future studies, all 4 end points of the Quadruple Aim should be represented to aid implementation in health care systems. We believe that further research is worth conducting to strengthen the evidence regarding wearables in chronic disease management.

### Conclusions

Our systematic review did not find a clear role of wearables in improving health care outcomes in chronic disease. However, wearables are becoming increasingly popular within the community, and as research and development in wearable technology progress, it is anticipated that these devices will play an increasing role in supporting healthy lifestyle modifications for their users. More research is required to ascertain a clear causality between wearables and health care outcomes, as defined by the Quadruple Aim, for people with chronic disease. As the evidence base for the use of wearables in chronic disease management is strengthened, further challenges will need to be overcome to allow widespread adoption in the health care setting, including stringent regulatory approval, data privacy and confidentiality, and software accuracy.

## Appendix

Multimedia Appendix 1 PRISMA (Preferred Reporting Items for Systematic Reviews and Meta-Analyses) checklist.

Multimedia Appendix 2 Search strings for systematic review.

Multimedia Appendix 3 Risk of bias assessment for randomized studies.

Multimedia Appendix 4 Risk of bias assessment for nonrandomized studies.

Multimedia Appendix 5 Summary of articles included in systematic review classified by disease group.

Multimedia Appendix 6 Outcome measures within included studies.

## Abbreviations

6MWT: 6-minute walk test
AR: augmented reality
COPD: chronic obstructive pulmonary disease
HbA_1c_: hemoglobin A_1c_
IMU: inertial measurement unit
MeSH: Medical Subject Headings
MWD: maximal walking distance
PR: pulmonary rehabilitation
PRISMA: Preferred Reporting Items for Systematic Reviews and Meta-Analyses
PROSPERO: International Prospective Register for Systematic Reviews
RCT: randomized controlled trial
VO_2_ max: maximal oxygen consumption
VR: virtual reality
