# Supplementary figures and images for: The Influence of Wearables on Health Care Outcomes in Chronic Disease: Systematic Review
Source: J Med Internet Res. 2022 Jul 1;24(7):e36690. doi: 10.2196/36690 (PMC9288104; doi:10.2196/36690)

## Multimedia Appendix 1: PRISMA Checklist

##
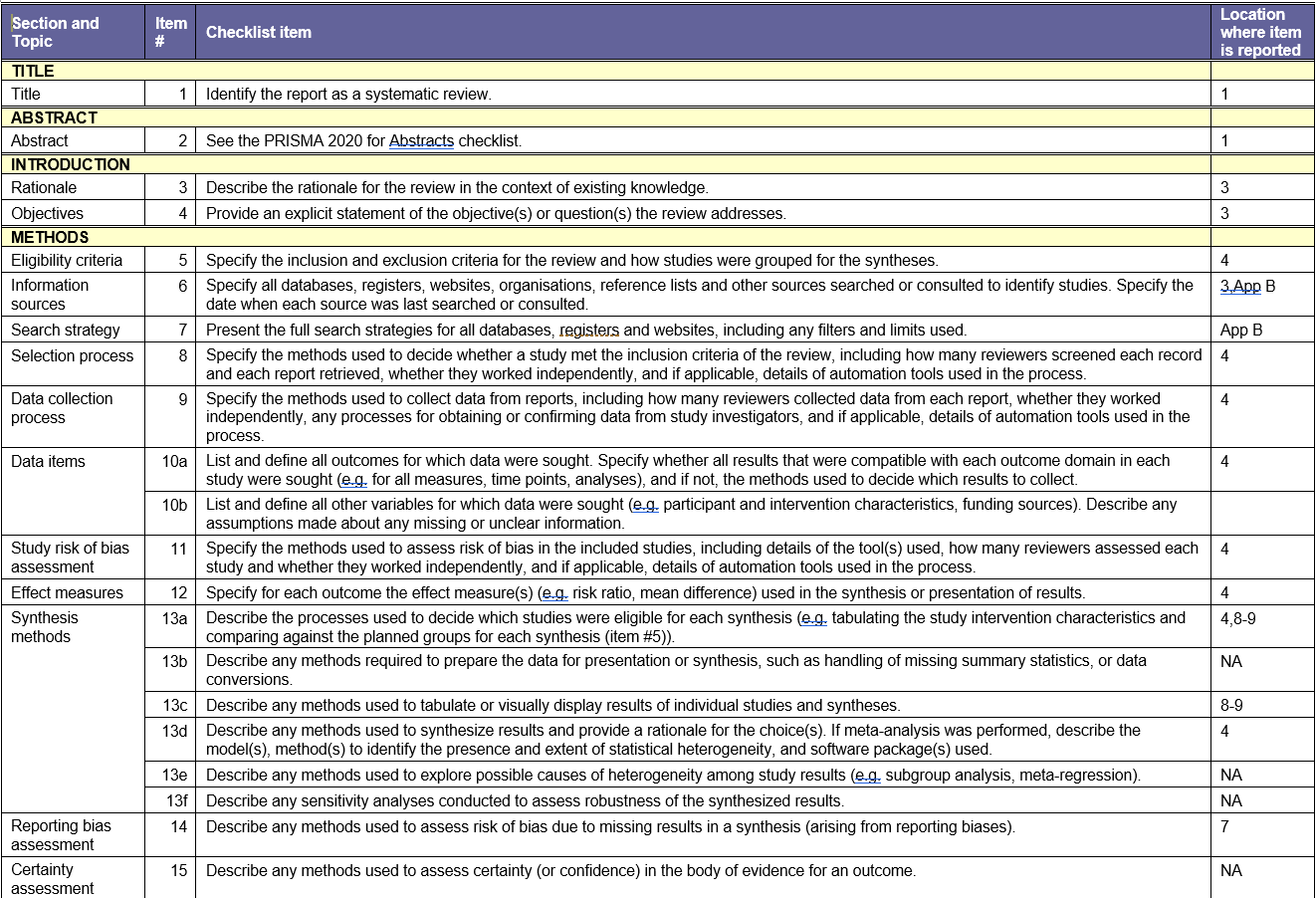


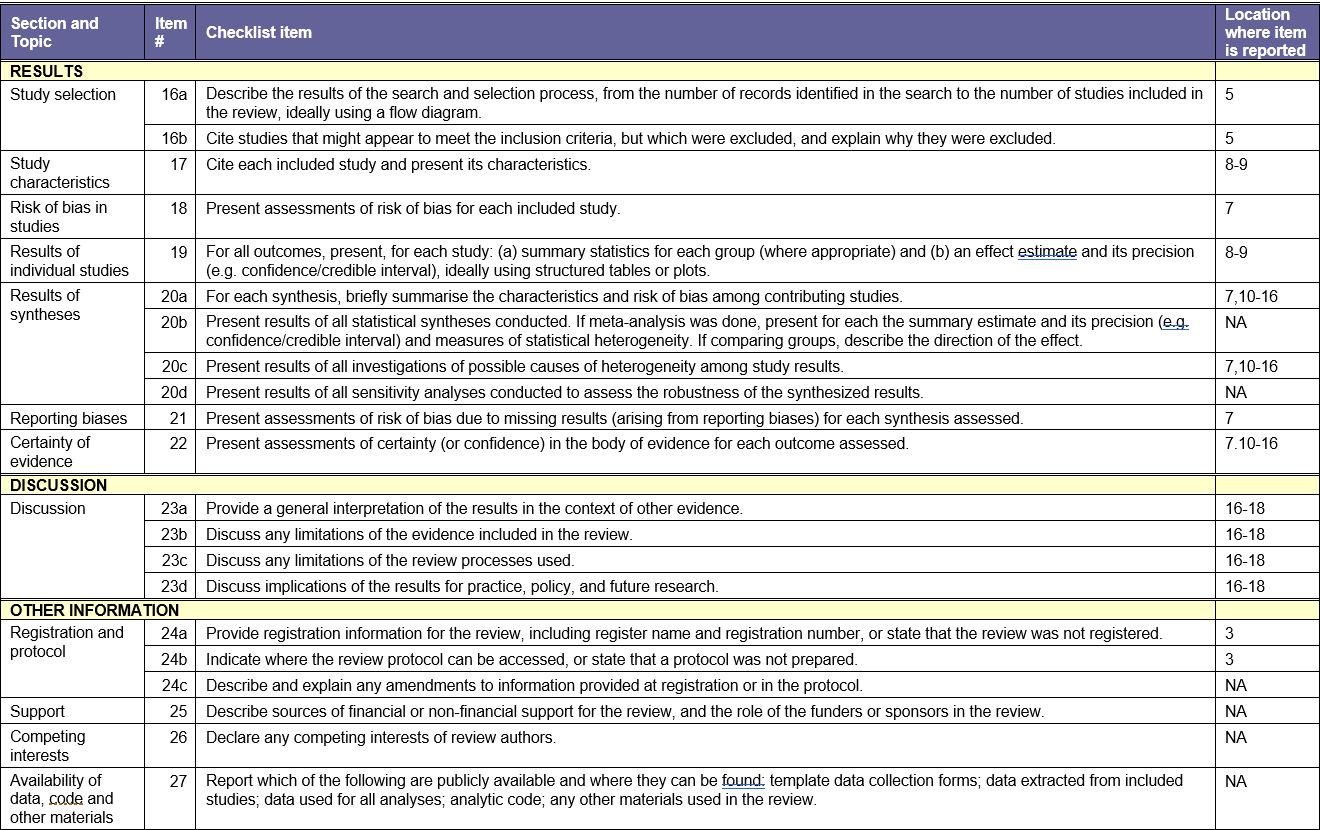

Supplement: Multimedia Appendix 1 [file jmir_v24i7e36690_app1.docx]
