# Supplementary material for: The Influence of Wearables on Health Care Outcomes in Chronic Disease: Systematic Review
Source: J Med Internet Res. 2022 Jul 1;24(7):e36690. doi: 10.2196/36690 (PMC9288104; doi:10.2196/36690)
Supplement: Multimedia Appendix 2 [file jmir_v24i7e36690_app2.docx]

## Multimedia Appendix 2: Search strings for systematic review

- **PubMed:** ("Chronic Disease"[Mesh] OR "Asthma"[Mesh] OR "Pulmonary Disease, Chronic Obstructive"[Mesh] OR "Emphysema"[Mesh] OR "Bronchitis, Chronic"[Mesh] OR "Diabetes Mellitus"[Mesh] OR "Osteoarthritis"[Mesh] OR "Arthritis, Rheumatoid"[Mesh] OR "Hypertension"[Mesh] OR "Pulmonary Fibrosis"[Mesh] OR "Lung Diseases, Interstitial"[Mesh] OR "Heart Failure"[Mesh] OR "Myocardial Ischemia"[Mesh] OR "Angina Pectoris"[Mesh] OR "Endocarditis"[Mesh] OR "Atrial Fibrillation"[Mesh] OR "Arrhythmias, Cardiac"[Mesh] OR "Bronchiectasis"[Mesh] OR "Lupus Erythematosus, Systemic"[Mesh] OR "Renal Insufficiency, Chronic"[Mesh] OR "Glomerulonephritis"[Mesh] OR "Nephrotic Syndrome"[Mesh] OR "Inflammatory Bowel Diseases"[Mesh] OR "Crohn Disease"[Mesh] OR "Colitis, Ulcerative"[Mesh] OR "Liver Cirrhosis"[Mesh] OR "Cholelithiasis"[Mesh] OR "Choledocholithiasis"[Mesh] OR "Pancreatitis, Chronic"[Mesh] OR "Stroke"[Mesh] OR "Hemorrhagic Stroke"[Mesh] OR "Ischemic Stroke"[Mesh] OR "Brain Ischemia"[Mesh] OR "Embolic Stroke"[Mesh] OR "Multiple Sclerosis"[Mesh] OR "Motor Neuron Disease"[Mesh] OR "Amyotrophic Lateral Sclerosis"[Mesh] OR "Bulbar Palsy, Progressive"[Mesh] OR "Dementia"[Mesh] OR "Huntington Disease"[Mesh] OR "Parkinson Disease"[Mesh] OR "chronic disease"[Title/Abstract] OR asthma[Title/Abstract] OR "Chronic obstructive pulmonary disease"[Title/Abstract] OR COPD[Title/Abstract] OR emphysema[Title/Abstract] OR "chronic bronchitis"[Title/Abstract] OR "Diabetes Mellitus"[Title/Abstract] OR "Osteoarthritis"[Title/Abstract] OR "Rheumatoid Arthritis"[Title/Abstract] OR "Seronegative Arthr*"[Title/Abstract] OR Hypertension[Title/Abstract] OR "high blood pressure"[Title/Abstract] OR "Pulmonary fibrosis"[Title/Abstract] OR "interstitial lung disease"[Title/Abstract] OR "Occupational lung disease"[Title/Abstract] OR "Heart failure"[Title/Abstract] OR "congestive cardiac failure"[Title/Abstract] OR "Ischaemic heart disease"[Title/Abstract] OR angina*[Title/Abstract] OR endocarditis[Title/Abstract] OR "atrial fibrillation"[Title/Abstract] OR arrhythmia[Title/Abstract] OR bronchiectasis[Title/Abstract] OR "systemic lupus erythematosus"[Title/Abstract] OR lupus[Title/Abstract] OR "chronic kidney disease"[Title/Abstract] OR glomerulonephritis[Title/Abstract] OR "nephrotic syndrome"[Title/Abstract] OR "nephritic syndrome"[Title/Abstract] OR "inflammatory bowel disease"[Title/Abstract] OR "Crohn disease"[Title/Abstract] OR "Crohns disease"[Title/Abstract] OR "ulcerative colitis"[Title/Abstract] OR cirrhosis[Title/Abstract] OR cholelithiasis[Title/Abstract] OR choledocholithiasis[Title/Abstract] OR "chronic pancreatitis"[Title/Abstract] OR malignancy[Title/Abstract] OR stroke[Title/Abstract] OR "ischaemic stroke"[Title/Abstract] OR "cerebrovascular accident"[Title/Abstract] OR "haemorrhagic stroke"[Title/Abstract] OR "cardioembolic stroke"[Title/Abstract] OR parkinson* OR "multiple sclerosis"[Title/Abstract] OR "motor neuron disease"[Title/Abstract] OR "amyotrophic lateral sclerosis"[Title/Abstract] OR "bulbar palsy"[Title/Abstract] OR dementia[Title/Abstract] OR "huntington chorea"[Title/Abstract] OR "huntington disease"[Title/Abstract] OR huntington*[Title/Abstract]) **AND** ("Wearable Electronic Devices"[Mesh] OR "Fitness Trackers"[Mesh] OR wearable*[Title/Abstract]) **AND** (healthcare OR outcome*)
- **Web of Science: (((ALL=(chronic*) ) AND ALL=((illness*)OR(disease*))) AND ((TS=smartwatch*) ) OR TS=(activity tracker*) OR TS=(wearable tech*)) AND ALL=((healthcare*) AND (outcome*))**
- **EMBASE:** ('chronic disease'/exp OR 'chronic disease':ti,ab,kw OR 'asthma'/exp OR asthma:ti,ab,kw OR 'chronic obstructive pulmonary disease'/exp OR 'chronic obstructive pulmonary disease':ti,ab,kw OR copd:ti,ab,kw OR 'emphysema'/exp OR emphysema:ti,ab,kw OR 'chronic bronchitis'/exp OR 'chronic bronchitis':ti,ab,kw OR 'diabetes mellitus'/exp OR 'diabetes mellitus':ti,ab,kw OR 'osteoarthritis'/exp OR 'osteoarthritis':ti,ab,kw OR 'rheumatoid arthritis'/exp OR 'rheumatoid arthritis':ti,ab,kw OR 'seronegative arthr*':ti,ab,kw OR 'hypertension'/exp OR hypertension:ti,ab,kw OR 'pulmonary fibrosis'/exp OR 'pulmonary fibrosis':ti,ab,kw OR 'interstitial lung disease'/exp OR 'interstitial lung disease':ti,ab,kw OR 'occupational lung disease'/exp OR 'occupational lung disease':ti,ab,kw OR 'heart failure'/exp OR 'heart failure':ti,ab,kw OR 'congestive cardiac failure'/exp OR 'congestive cardiac failure':ti,ab,kw OR 'ischaemic heart disease'/exp OR 'ischaemic heart disease':ti,ab,kw OR angina*:ti,ab,kw OR 'endocarditis'/exp OR endocarditis:ti,ab,kw OR 'atrial fibrillation'/exp OR 'atrial fibrillation':ti,ab,kw OR 'arrhythmia'/exp OR arrhythmia:ti,ab,kw OR 'bronchiectasis'/exp OR bronchiectasis:ti,ab,kw OR 'systemic lupus erythematosus'/exp OR 'systemic lupus erythematosus':ti,ab,kw OR 'lupus'/exp OR lupus:ti,ab,kw OR 'chronic kidney disease'/exp OR 'chronic kidney disease':ti,ab,kw OR 'glomerulonephritis'/exp OR glomerulonephritis:ti,ab,kw OR 'nephrotic syndrome'/exp OR 'nephrotic syndrome':ti,ab,kw OR 'nephritic syndrome'/exp OR 'nephritic syndrome':ti,ab,kw OR 'inflammatory bowel disease'/exp OR 'inflammatory bowel disease':ti,ab,kw OR 'crohn* disease':ti,ab,kw OR 'ulcerative colitis'/exp OR 'ulcerative colitis':ti,ab,kw OR 'cirrhosis'/exp OR cirrhosis:ti,ab,kw OR 'cholelithiasis'/exp OR cholelithiasis:ti,ab,kw OR 'choledocholithiasis'/exp OR choledocholithiasis:ti,ab,kw OR 'chronic pancreatitis'/exp OR 'chronic pancreatitis':ti,ab,kw OR 'malignancy'/exp OR malignancy:ti,ab,kw OR 'stroke'/exp OR stroke:ti,ab,kw OR 'ischaemic stroke'/exp OR 'ischaemic stroke':ti,ab,kw OR 'cerebrovascular accident'/exp OR 'cerebrovascular accident':ti,ab,kw OR 'haemorrhagic stroke'/exp OR 'haemorrhagic stroke':ti,ab,kw OR 'cardioembolic stroke'/exp OR 'cardioembolic stroke':ti,ab,kw OR 'multiple sclerosis'/exp OR 'multiple sclerosis':ti,ab,kw OR 'motor neuron disease'/exp OR 'motor neuron disease':ti,ab,kw OR 'amyotrophic lateral sclerosis'/exp OR 'amyotrophic lateral sclerosis':ti,ab,kw OR 'bulbar palsy'/exp OR 'bulbar palsy':ti,ab,kw OR 'dementia'/exp OR dementia:ti,ab,kw OR 'huntington chorea'/exp OR huntington*:ti,ab,kw OR 'angina pectoris'/exp OR 'parkinson disease'/exp OR 'crohn disease'/exp OR parkinson*:ti,ab,kw OR crohn*:ti,ab,kw) **AND** ('wearable computer'/exp OR 'wearable device'/exp OR wearable:ti,ab,kw OR 'activity tracker'/exp) **AND** (healthcare* OR outcome*
- **Scopus:** TITLE-ABS-KEY ( ( "Chronic Disease"  OR  asthma  OR  " Chronic Obstructive Pulmonary Disease"  OR  emphysema  OR  "Chronic Bronchitis"  OR  "Diabetes Mellitus"  OR  osteoarthritis  OR  " Rheumatoid Arthritis"  OR  hypertension  OR  "Pulmonary Fibrosis"  OR  "Interstitial Lung Disease*"  OR  "Heart Failure"  OR  "Myocardial Ischemia"  OR  angina*  OR  endocarditis  OR  "Atrial Fibrillation"  OR  "Cardiac Arrhythmias"  OR  "Bronchiectasis"  OR  "Systemic Lupus Erythematosus"  OR  glomerulonephritis  OR  "Nephrotic Syndrome"  OR  "Inflammatory Bowel Disease*"  OR  "Crohn* Disease"  OR  "Ulcerative Colitis"  OR  ( liver  W/3  cirrhosis )  OR  "Liver Cirrhosis"  OR  cholelithiasis  OR  choledocholithiasis  OR  "Chronic Pancreatitis"  OR  stroke  OR  "Brain Ischemia"  OR  "Progressive Multiple Sclerosis"  [mesh]  OR  "Motor Neuron Disease"  OR  "Amyotrophic Lateral Sclerosis"  OR  "Bulbar Palsy"  OR  dementia  OR  "Huntington Disease"  OR  "Parkinson Disease"  OR  copd  OR  "Seronegative Arthr*"  OR  "high blood pressure"  OR  "interstitial lung disease"  OR  "Occupational lung disease"  OR  "congestive cardiac failure"  OR  "Ischaemic heart disease"  OR  arrhythmia  OR  lupus  OR  "chronic kidney disease"  OR  "nephritic syndrome"  OR  "Crohns disease"  OR  cirrhosis  OR  cholelithiasis  OR  choledocholithiasis  OR  malignancy  OR  "cerebrovascular accident"  OR  parkinson*  OR  "multiple sclerosis"  OR  "bulbar palsy"  OR  huntington* )  **AND**  ( "Wearable Electronic Devices"  OR  "Fitness Tracker*"  OR  wearable* )  **AND**  ( healthcare  OR  outcome* ) )
- **CINAHL:** (wearable tech* OR activity tracker*) **AND** (chronic* OR illness*)
- **Cochrane:** ((wearable*):ti,ab,kw OR (activity tracker*):ti,ab,kw) **AND** ((chronic* OR illness* OR (‘Asthma’ OR (‘Chronic Obstructive Pulmonary Disease’ OR ‘COPD’ OR ‘emphysema’ OR ‘chronic bronchitis’) OR ‘Diabetes Mellitus’ OR ‘Osteoarthritis’ OR ‘Rheumatoid Arthritis’ OR ‘Seronegative Arthr*’ OR ‘Hypertension’ OR ‘Pulmonary fibrosis’ OR ‘Interstitial lung disease’ OR ‘Occupational lung disease’ OR (‘Heart failure’ OR ‘heart failure*’ OR ‘congestive cardiac failure’ OR ‘HFpEF’ OR ‘HFrEF’) OR ‘Ischaemic heart disease’ OR ‘angina*’ OR ‘endocarditis’ OR ‘atrial fibrillation’ OR ‘arrhythmia’ OR ‘bronchiectasis’ OR (‘systemic lupus erythematosus’ OR ‘SLE’ OR ‘lupus’) OR ‘chronic kidney disease’ OR ‘glomerulonephritis’ OR ‘nephrotic syndrome’ OR ‘nephritic syndrome’ OR ‘inflammatory bowel disease’ OR ‘Crohn* disease’ OR ‘ulcerative colitis’ OR ‘cirrhosis’ OR ‘cholelithiasis’ OR ‘choledocholithiasis’ OR ‘chronic pancreatitis’ OR ‘malignancy’ OR (‘stroke’ OR ‘ischaemic stroke’ OR ‘cerebrovascular accident’ OR ‘haemorrhagic stroke’ OR ‘cardioembolic stroke’) OR ‘parkinson*’ OR ‘multiple sclerosis’ OR (‘motor neuron disease’ OR ‘amyotrophic lateral sclerosis’ OR ‘bulbar palsy’) OR ‘dementia’ OR ‘huntington*’)):ti,ab,kw) **AND** (‘healthcare*’ or ‘outcome*’)
