# Supplementary material for: The Influence of Wearables on Health Care Outcomes in Chronic Disease: Systematic Review
Source: J Med Internet Res. 2022 Jul 1;24(7):e36690. doi: 10.2196/36690 (PMC9288104; doi:10.2196/36690)
Supplement: Multimedia Appendix 3 [file jmir_v24i7e36690_app3.docx]

## **Multimedia Appendix 3.** Risk of Bias assessment for randomised studies

| **Author, Year** | **Sequence generation** | **Allocation concealment** | **Blinding of participants and personnel** | **Blinding of outcome assessment** | **Incomplete outcome data** | **Selective reporting** | **Other sources of bias** |
| --- | --- | --- | --- | --- | --- | --- | --- |
| *Abbott et al, 2019 [50]* | - | - | + | - | - | - | - |
| *Amorim et al, 2019 [48]* | ? | - | + | - | - | - | - |
| *Austin et al, 2020 [43]* | - | ? | + | - | - | - | - |
| *Blitz et al, 2018 [34]* | - | - | + | - | - | - | - |
| *Bortone et al, 2020 [32]* | - | - | + | ? | - | - | - |
| *Carpinella et al, 2017 [61]* | - | - | + | - | - | - | - |
| *Chen et al, 2020 [45]* | - | - | + | - | - | - | - |
| *Frias et al, 2017 [49]* | + | - | + | - | - | - | ? |
| *Garcia et al, 2021 [40]* | - | - | - | - | - | - | - |
| *Heldman et al, 2017 [57]* | - | - | + | - | - | - | - |
| *Katz et al, 2018 [44]* | ? | - | + | - | - | - | - |
| *Kim et al, 2019 [35]* | ? | - | + | - | - | - | - |
| *Kooiman et al, 2018 [46]* | - | - | + | ? | - | - | - |
| *Lang et al, 2021 [36]* | - | - | + | - | - | - | - |
| *Li et al, 2020 [39]* | - | + | + | - | - | - | - |
| *Li et al, 2020 [41]* | - | - | + | - | - | - | - |
| *Lin et al, 2018 [47]* | - | ? | + | - | - | - | - |
| *Lystrup et al, 2020 [52]* | - | - | + | - | - | - | - |
| *Maddison et al, 2019 [55]* | - | - | + | - | - | - | - |
| *Normahani et al, 2018 [59]* | - | - | + | - | - | - | - |
| *Smith et al, 2019 [53]* | - | - | + | - | - | - | - |
| *Takahashi et al, 2016 [51]* | - | - | + | - | - | - | - |
| *Taylor et al, 2021 [38]* | - | - | + | - | - | - | - |
| *Widyastuti et al, 2018 [54]* | ? | ? | + | - | - | - | - |

(+) – high risk of bias; (-) – low risk of bias; (?) – unsure
