# Supplementary material for: The Influence of Wearables on Health Care Outcomes in Chronic Disease: Systematic Review
Source: J Med Internet Res. 2022 Jul 1;24(7):e36690. doi: 10.2196/36690 (PMC9288104; doi:10.2196/36690)
Supplement: Multimedia Appendix 4 [file jmir_v24i7e36690_app4.docx]

## **Multimedia Appendix 4.** Risk of bias assessment for non-randomised studies

| **Author, Year** | **Confounding bias** | **Selection bias** | **Classification of Interventions bias** | **Deviation from intended intervention bias** | **Missing data bias** | **Measurement of outcomes bias** | **Selection of result bias** |
| --- | --- | --- | --- | --- | --- | --- | --- |
| *Huh et al, 2019 [37]* | - | - | - | - | - | - | - |
| *Stuart et al, 2020 [42]* | - | - | - | - | - | - | - |
| *Ward et al, 2020 [56]* | - | - | - | - | - | - | - |
| *Takahashi et al, 2019 [64]* | - | ? | - | ? | - | - | ? |
| *Tunur et al, 2019 [74]* | - | ? | - | - | - | - | ? |
| *Zaslavsky et al, 2019 [60]* | - | - | - | - | - | - | - |

^(+) – high risk of bias; (-) – low risk of bias; (?) – unsure^
