# Supplementary material for: The Influence of Wearables on Health Care Outcomes in Chronic Disease: Systematic Review
Source: J Med Internet Res. 2022 Jul 1;24(7):e36690. doi: 10.2196/36690 (PMC9288104; doi:10.2196/36690)
Supplement: Multimedia Appendix 5 [file jmir_v24i7e36690_app5.docx]

## **Multimedia Appendix 5.** Summary of articles included in systematic review classified by disease group

| **Chronic Disease** | **Author, Year** | **Sample Size** | **Setting** | **Intervention (Wearable)** | **Control**  **(if any)** | **Primary Outcome** | **Unit of Measurement** | **Effect (vs Control if any)** |
| --- | --- | --- | --- | --- | --- | --- | --- | --- |
| **Stroke and Neurology** | | | | | | | | |
| *Cerebral palsy & developmental dyspraxia* | Bortone *et al* 2020 [32] | 8 | Rehabilitation centre | VR-assisted rehabilitation | Conventional rehabilitation | Degree of upper limb functional disability | 1) 9-hole peg test (right and left hand, time to completion)  2) Unified Clinical Index – Score (%) | None |
| *Chronic neuropathic pain (post-spinal cord injury)* | Austin *et al* 2020 [43] | 17 | Specialist outpatients | 3D head-mounted display VR (Oculus Rift) | Sham VR – 2D screen | Neuropathic Pain Score | Numerical pain rating scale; range 0-10 | Positive |
| *Stroke* | Lin *et al* 2018 [47] | 20 | Rehabilitation centre | Wearable rehabilitation monitoring system | Conventional rehabilitation | Upper limb neurological recovery | Fugl-Meyer Assessment | Positive |
| *Parkinson’s Disease* | Carpinella *et al* 2017 [61] | 42 | Rehabilitation centre | Wearable 6-inertial sensor system | Conventional physiotherapy | Balance & self-selected gait speed | 1) Berg Balance Scale (BBS); range 0-56  2) 10-minute walk test (10MWT) | Positive |
| *Parkinson’s Disease* | Heldman *et al* 2017 [57] | 18 | Specialist outpatients | Motion sensor-based monitoring system with neurologist advice from data | System without neurologist accessing data | Parkinson’s Disease symptom severity | Unified Parkinson’s Disease Rating Scale (UPDRS); range 0-199 | None |
| *Parkinson’s Disease* | Tunur *et al* 2020 [74] | 7 | Specialist outpatients | Google Glass + AR-based dance intervention | No intervention | Motor Assessment | Multiple | None |
| *Progressive Multiple Sclerosis* | Stuart *et al* 2020 [42] | 56 | Specialist outpatients | SenseWear armband | No intervention | Physical disability and brain volume (MRI) | Composite remote activity monitoring (RAM) score | Positive |
| **Rheumatology and Musculoskeletal** | | | | | | | | |
| *Chronic lower back pain* | Garcia *et al* 2021 [40] | 188 | Community | Skills-based interactive 3D VR head-mounted system | Sham VR | Average pain intensity | Defence and Veterans Pain Rating Scale; range 0-10 | Positive |
| *Chronic lower back pain* | Amorim *et al* 2019 [48] | 68 | Community | Fitbit activity tracker + app (IMPACT) | Information booklet & education | Care seeking episodes | Number | None |
| *Chronic lower back pain* | Lang *et al* 2021 [36] | 174 | Community | Pedometer (Yamax DigiWalker CW-701) | Usual care | Perceived disability | Oswestry Disability Index | None |
| *Juvenile Idiopathic Arthritis (JIA)* | Blitz *et al* 2018 [34] | 27 | Community | Pedometer (Omron HJ-720ITC) + “Walk with Ease” seminar | Pedometer only | 6MWT | Metres | Positive |
| *Knee Osteoarthritis* | Smith *et al* 2019 [53] | 60 | Specialist clinic | Fitbit Flex + app; 16-week exercise program using Fitbit data | 16-week exercise program only | 6MWT | Metres | None |
| *Osteoarthritis* | Zaslavsky *et al* 2019 [60] | 24 | Community | Fitbit Charge 2 + personalised texts based on tracking data | No intervention | Sleep Quality | 1) Insomnia Severity Index (ISI)  2) Acceptance of Sleep Difficulties (ASD) Index  3) Wrist actigraphy | Positive |
| *Rheumatoid Arthritis* | Katz *et al* 2018 [44] | 96 | Specialist clinic | 1) Pedometer (Jawbone Up) & step targets  2) Pedometer only | Usual care (without pedometer) | Fatigue | PROMIS Fatigue Short Form 7a | Positive |
| *Rheumatoid Arthritis & Systemic Lupus Erythematosus* | Li *et al* 2020 [41] | 118 | Community | Fitbit Flex 2 + physiotherapy input based on activity tracker | Usual care | MVPA time | Minutes/day | None |
| **Respiratory** | | | | | | | | |
| *COPD* | Ward *et al* 2021 [56] | 30 | Rehabilitation outpatients | Pedometer (Fitbit Zip) + exercise prescription based on stepcount | No intervention | Use of activity monitor to walk at the prescribed intensity | Adherence to prescribed cadence (%) | Positive |
| *COPD* | Widyastuti *et al* 2018 [54] | 40 | Specialist outpatients | Pedometer (Omron HJ-321) + activity logbooks | Pulmonary rehabilitation | 6MWT | Metres | None |
| *Obstructive Sleep Apnoea* | Kim *et al*  2019 [35] | 60 | Specialist clinic | Samsung Charm fitness tracker + app (MyHealthKeeper) | 1) App only  2) No app or wearable | BMI | Kg/m^2^ | Positive |
| **Cardiology** | | | | | | | | |
| *Ischaemic heart disease* | Maddison et al 2019 [55] | 162 | Specialist clinic, rehabilitation centres | Chest-worn sensor (BioHarness 3) + app guiding home-based rehabilitation | Centre-based rehabilitation | VO_2_ max | ml/kg/min | None |
| **Endocrine** | | | | | | | | |
| *Diabetic foot ulceration* | Abbott *et al* 2019 [50] | 58 | Community | SurroSense Rx – intelligent insole with feedback | SurroSense Rx without feedback | Foot ulceration | Foot ulcer recurrence (n=) | None |
| *Type 2 Diabetes Mellitus* | Lystrup *et al* 2020 [52] | 120 | Military medical facility | Fitbit Charge + WhatsApp group-based motivation | Fitbit Charge only | Weight | Lbs | None |
| *Type 2 Diabetes Mellitus* | Kooiman *et al* 2018 [46] | 72 | Community | Pedometer (Fitbit Zip) + access to online self-tracking system | Usual care | HbA1c | Percentage and mmol/mol | None |
| *Type 2 Diabetes Mellitus and Hypertension* | Frias *et al* 2017 [49] | 118 | Primary care outpatients | Ingestible medication sensor & smart patch | Usual care | Change in systolic BP at 4 weeks | mmHg | Positive |
| **Metabolic Syndrome and Obesity** | | | | | | | | |
| *Metabolic Syndrome* | Huh *et al* 2019 [37] | 52 | Community | Pedometer (Coffee WALKIE) + app | No intervention | Resolution of metabolic syndrome | Number | Positive |
| *Obesity* | Takahashi *et al* 2016 [51] | 130 | Community | Pedometer (Omron HJ-112) + goal setting | Usual care | Step count/day | Number | None |
| *Obesity* | Takahashi *et al* 2019 [64] | 130 | Community | Pedometer (Omron HJ-112) + goal setting | Usual care | Weight loss | Participants achieving weight loss ≥5% across 4 months (n;%) | None |
| **Other** | | | | | | | | |
| *Chronic kidney disease (CKD)* | Li *et al* 2020 [39] | 60 | Specialist outpatients | Heart Rate Smart Wristband – GSH405-B6 + WowGoHealth app | Usual care | Self-efficacy & self-management | Self-efficacy questionnaire (internally validated, range 0-200) | Positive |
| *End-stage liver cirrhosis* | Chen *et al* 2020 [45] | 20 | Specialist outpatients | Fitbit Charge HR + home-based physical activity program | Dietary modification only | 6MWT | Metres | Positive |
| *Peripheral Vascular Disease* | Normahani *et al* 2018 [59] | 37 | Community | Nike^+^ FuelBand | Usual care | Maximum walking distance (MWD) | Metres | Positive |
| *Chronic Disease (multiple)* | Taylor *et al* 2021 [38] | 450 | Community | Pedometer + web-based exercise program | Usual care | MVPA time | Number of 10-minute bouts | None |
