# Supplementary material for: The Influence of Wearables on Health Care Outcomes in Chronic Disease: Systematic Review
Source: J Med Internet Res. 2022 Jul 1;24(7):e36690. doi: 10.2196/36690 (PMC9288104; doi:10.2196/36690)
Supplement: Multimedia Appendix 6 [file jmir_v24i7e36690_app6.docx]

## Multimedia Appendix 6. Outcome measures within included studies

| **Quadruple Aim** | **Author, Year** | **Outcome Measure** | **Primary/Secondary/Exploratory** |
| --- | --- | --- | --- |
| Clinician Experience | Carpinella 2017 | Satisfaction of clinicians with system | Secondary |
|  | Heldman 2017 | Time spent in consultation | Secondary |
| Cost | Maddison 2019 | Cost of intervention | Secondary |
|  | Widyastuti 2018 | Cost of intervention | Secondary |
| Healthcare Outcome | Blitz 2018 | Active joint count | Secondary |
|  | Lin 2018 | Active range of motion | Secondary |
|  | Carpinella 2017 | Activities specific balance confidence scale | Secondary |
|  | Amorim 2019 | Activity limitation | Secondary |
|  | Lang 2021 | Adherence to walking intervention | Secondary |
|  | Ward 2021 | Adherence to walking intervention | Primary |
|  | Kooiman 2018 | Advanced glycosylation end products | Secondary |
|  | Maddison 2019 | Adverse events | Secondary |
|  | Carpinella 2017 | Axial stability/sway | Secondary |
|  | Lang 2021 | Back beliefs | Secondary |
|  | Tunur 2019 | Balance and functional mobility | Secondary |
|  | Carpinella 2017 | Balance and self-selected gait speed | Primary |
|  | Tunur 2019 | Balance confidence | Secondary |
|  | Lystrup 2020 | Blood pressure | Secondary |
|  | Maddison 2019 | Blood pressure | Secondary |
|  | Widyastuti 2018 | BODE index | Secondary |
|  | Maddison 2019 | Body anthropometry | Secondary |
|  | Smith 2019 | Body anthropomorphics/vitals | Secondary |
|  | Li 2020 (CKD) | Body composition | Secondary |
|  | Blitz 2018 | Body mass index | Secondary |
|  | Stuart 2020 | Brain volume | Secondary |
|  | Abbott 2019 | Callus severity | Secondary |
| Healthcare Outcome | Huh 2019 | Calorie expenditure | Primary |
|  | Amorim 2019 | Care seeking episodes | Primary |
|  | Frias 2017 | Change in diastolic blood pressure | Secondary |
|  | Frias 2017 | Change in fasting plasma glucose | Secondary |
|  | Frias 2017 | Change in HbA1c | Secondary |
|  | Frias 2017 | Change in LDL using digital atorvastatin | Exploratory |
|  | Frias 2017 | Change in systolic blood pressure (wk 12) | Secondary |
|  | Frias 2017 | Change in systolic blood pressure (wk 4) | Primary |
|  | Kooiman 2018 | Change in use of medication | Secondary |
|  | Heldman 2017 | Changes in dopaminergic medication | Secondary |
|  | Ward 2021 | Chronic disease emotional functioning | Secondary |
|  | Ward 2021 | Chronic disease mastery | Secondary |
|  | Widyastuti 2018 | COPD Assessment Tool | Secondary |
|  | Li 2020 (CKD) | Creatinine/estimated glomerular filtration rate | Secondary |
|  | Austin 2021 | Depression and anxiety | Secondary |
|  | Katz 2018 | Depression/Anxiety | Secondary |
|  | Li 2020 | Depression/Anxiety | Secondary |
|  | Taylor 2020 | Depression/Anxiety | Secondary |
|  | Lin 2018 | Deviation angle of limb | Secondary |
|  | Bortone 2020 | Disability | Primary |
|  | Lang 2021 | Disability | Primary |
|  | Katz 2018 | Disease activity | Secondary |
|  | Carpinella 2017 | Disease severity | Secondary |
|  | Heldman 2017 | Disease severity | Secondary |
|  | Normahani 2018 | Distance to claudication | Secondary |
|  | Ward 2021 | Dyspnoea | Secondary |
|  | Widyastuti 2018 | Dyspnoea | Secondary |
|  | Blitz 2018 | Estimated time walking | Secondary |
|  | Maddison 2019 | Exercise adherence | Secondary |
|  | Maddison 2019 | Exercise-related motivation | Secondary |
|  | Maddison 2019 | Fasting lipids and glucose levels | Secondary |
| Healthcare Outcome | Katz 2018 | Fatigue | Primary |
|  | Li 2020 | Fatigue | Secondary |
|  | Ward 2021 | Fatigue | Secondary |
|  | Lang 2021 | Fear-Avoidance Beliefs | Secondary |
|  | Abbott 2019 | Foot ulcer recurrence | Primary |
|  | Carpinella 2017 | Freezing of gait questionnaire | Secondary |
|  | Katz 2018 | Functional impairment | Secondary |
|  | Takahashi 2016 | Gait speed | Secondary |
|  | Takahashi 2016 | Grip strength | Secondary |
|  | Kooiman 2018 | HbA1c | Primary |
|  | Lystrup 2020 | HbA1c | Secondary |
|  | Blitz 2018 | Heart rate pre- and post six minute walk test | Secondary |
|  | Ward 2021 | Incremental shuttle walking test | Secondary |
|  | Normahani 2018 | Maximum walking distance | Primary |
|  | Li 2020 | Mean time in purposeful activity | Secondary |
|  | Li 2020 | Mean time in sedentary behaviour | Secondary |
|  | Frias 2017 | Medication adherence | Secondary |
|  | Li 2020 | Moderate-to-vigorous physical activity time | Primary |
|  | Taylor 2020 | Moderate-to-vigorous physical activity time (episodes) | Primary |
|  | Tunur 2019 | One-leg stance | Secondary |
|  | Amorim 2019 | Pain | Secondary |
|  | Austin 2021 | Pain | Primary |
|  | Blitz 2018 | Pain | Secondary |
|  | Garcia 2021 | Pain | Primary |
|  | Katz 2018 | Pain | Secondary |
|  | Lang 2021 | Pain | Secondary |
| Healthcare Outcome | Li 2020 | Pain | Secondary |
|  | Garcia 2021 | Pain interference with activity | Secondary |
|  | Garcia 2021 | Pain interference with mood | Secondary |
|  | Garcia 2021 | Pain interference with sleep | Secondary |
|  | Garcia 2021 | Pain interference with stress | Secondary |
|  | Carpinella 2017 | Parkinson's disease questionnaire-39 | Secondary |
|  | Garcia 2021 | Patient global impression of change | Secondary |
|  | Blitz 2018 | Perceived exertion (Borg Scale) | Secondary |
|  | Lang 2021 | Physical activity | Secondary |
|  | Chen 2020 | Physical fitness | Primary |
|  | Blitz 2018 | Physical function | Primary |
|  | Lystrup 2020 | Physical function | Secondary |
|  | Smith 2019 | Physical function | Primary |
|  | Widyastuti 2018 | Physical function | Primary |
|  | Garcia 2021 | Physical function and sleep disturbance | Secondary |
|  | Garcia 2021 | Prescription opiate and OTC analgesic use | Secondary |
|  | Frias 2017 | Proportion of participants at blood pressure goal | Secondary |
|  | Ward 2021 | Quadriceps maximal voluntary contraction | Secondary |
|  | Blitz 2018 | Quality of Life | Secondary |
|  | Lang 2021 | Quality of Life | Secondary |
|  | Li 2020 (CKD) | Quality of Life | Secondary |
|  | Maddison 2019 | Quality of Life | Secondary |
|  | Normahani 2018 | Quality of Life | Secondary |
|  | Smith 2019 | Quality of Life | Secondary |
|  | Taylor 2020 | Quality of Life | Secondary |
|  | Huh 2019 | Resolution of metabolic syndrome | Primary |
|  | Blitz 2018 | Self-efficacy | Secondary |
|  | Lang 2021 | Self-efficacy | Secondary |
|  | Li 2020 | Self-efficacy | Secondary |
|  | Li 2020 (CKD) | Self-efficiacy | Primary |
|  | Li 2020 (CKD) | Self-management | Secondary |
| Healthcare Outcome | Taylor 2020 | Self-reported achievement of activity goals | Secondary |
|  | Li 2020 | Self-reported habit index | Secondary |
|  | Smith 2019 | Self-reported knee function | Secondary |
|  | Taylor 2020 | Self-reported weekly minutes of moderate-to-vigorous physical activity | Secondary |
|  | Chen 2020 | Sickness impact profile score | Secondary |
|  | Chen 2020 | Skeletal muscle anthropometry | Secondary |
|  | Kim 2019 | Sleep | Secondary |
|  | Taylor 2020 | Sleep and sedentary behaviour time | Secondary |
|  | Zaslavsky 2019 | Sleep quality | Primary |
|  | Bortone 2020 | Smoothness of movement | Secondary |
|  | Frias 2017 | Step count | Secondary |
|  | Huh 2019 | Step count | Primary |
|  | Li 2020 | Step count | Secondary |
|  | Li 2020 (CKD) | Step count | Secondary |
|  | Lystrup 2020 | Step count | Secondary |
|  | Takahashi 2016 | Step count | Primary |
|  | Widyastuti 2018 | Step count | Secondary |
|  | Kooiman 2018 | Subjective health visual analog scale | Secondary |
|  | Abbott 2019 | Time to ulceration | Secondary |
|  | Carpinella 2017 | Timed up and go test | Secondary |
|  | Tunur 2019 | Timed up and go test | Secondary |
|  | Taylor 2020 | Total minutes of moderate-to-vigorous physical activity | Secondary |
|  | Lin 2018 | Upper limb neurological recovery | Primary |
|  | Stuart 2020 | Validation of remote monitoring system | Primary |
|  | Maddison 2019 | VO2 max | Primary |
|  | Kooiman 2018 | Waist-hip ratio | Secondary |
|  | Kooiman 2018 | Weight | Secondary |
|  | Lystrup 2020 | Weight | Primary |
|  | Kim 2019 | Weight/BMI | Primary |
| Patient Experience | Tunur 2019 | Acceptance of Google Glass | Primary |
|  | Li 2020 | Adherence | Secondary |
|  | Abbott 2019 | Adherence to wearing device | Secondary |
|  | Heldman 2017 | Compliance with wearable | Primary |
|  | Taylor 2020 | Engagement with intervention | Secondary |
|  | Garcia 2021 | Engagement with treatment | Secondary |
|  | Taylor 2020 | Experience of using intervention | Secondary |
|  | Austin 2021 | Presence during VR | Secondary |
|  | Blitz 2018 | Satisfaction with treatment | Secondary |
|  | Garcia 2021 | Satisfaction with treatment | Secondary |
|  | Taylor 2020 | Uptake of self-exercise referral scheme | Secondary |
|  | Heldman 2017 | Wearable usability | Secondary |
